# Cardiac Performance and Cardiopulmonary Fitness After Infection With SARS-CoV-2

**DOI:** 10.3389/fcvm.2022.871603

**Published:** 2022-05-13

**Authors:** Gregory Wood, Therese Stegeager Kirkevang, Jane Agergaard, Steffen Leth, Esben Søvsø Szocska Hansen, Christoffer Laustsen, Anders Hostrup Larsen, Henrik Kjærulf Jensen, Lars Jørgen Østergaard, Hans Erik Bøtker, Steen Hvitfeldt Poulsen, Won Yong Kim

**Affiliations:** ^1^Department of Cardiology, Aarhus University Hospital, Aarhus, Denmark; ^2^Department of Clinical Medicine, Aarhus University, Aarhus, Denmark; ^3^Department of Infectious Disease, Aarhus University Hospital, Aarhus, Denmark; ^4^Department of Clinical Medicine, Aarhus University, Aarhus, Denmark; ^5^Department of Infectious Diseases, Internal Medicine, Gødstrup Hospital, Herning, Denmark; ^6^The MR Research Centre, Department of Clinical Medicine, Aarhus University, Aarhus, Denmark

**Keywords:** CMR, echocardiography, long-COVID syndrome, COVID-19, recovery following COVID-19

## Abstract

**Aims:**

Persistent cardiac symptoms are an increasingly reported phenomenon following COVID-19. However, the underlying cause of cardiac symptoms is unknown. This study aimed to identify the underlying causes, if any, of these symptoms 1 year following acute COVID-19 infection.

**Methods and Results:**

22 individuals with persistent cardiac symptoms were prospectively investigated using echocardiography, cardiovascular magnetic resonance (CMR), 6-min walking test, cardio-pulmonary exercise testing and electrocardiography. A median of 382 days (IQR 368, 442) passed between diagnosis of COVID-19 and investigation. As a cohort their echocardiography, CMR, 6-min walking test and exercise testing results were within the normal ranges. There were no differences in left ventricular ejection fraction (61.45 ± 6.59 %), global longitudinal strain (19.80 ± 3.12 %) or tricuspid annular plane systolic excursion (24.96 ± 5.55 mm) as measured by echocardiography compared to a healthy control group. VO2 max (2045.00 ± 658.40 ml/min), % expected VO2 max (114.80 ± 23.08 %) and 6-minute distance walked (608.90 ± 54.51 m) exceeded that expected for the patient cohort, whilst Troponin I (5.59 ± 6.59 ng/l) and Nt-proBNP (88.18 ± 54.27 ng/l) were normal.

**Conclusion:**

Among a cohort of 22 patients with self-reported persistent cardiac symptoms, we identified no underlying cardiac disease or reduced cardiopulmonary fitness 1 year following COVID-19.

## Introduction

One of the major health issues being increasingly recognized following COVID-19 infection, is the high prevalence of ‘long-COVID' syndrome that develops in patients following resolution of the acute infection. This is defined by the Center for Disease Control as “a wide range of new, returning, or ongoing health problems people can experience four or more weeks after first being infected with the virus that causes COVID-19” (1). A recent study from our research group has indicated that 96% of admitted patients with COVID-19 experience at least one persistent symptom 3 months following the acute phase of the disease (2), with further evidence indicating that this falls to 68% and 49% at 6 and 12 months respectively (3).

A wide variety of symptoms have been reported with varying degrees of severity. Dyspnoea and chest pain are two of the more common symptoms that patients experience following recovery from the acute COVID-19 infection, affecting 88% and 76% of long COVID patients, respectively 5 months following infection (4). Previous studies have speculated that one of the main causes of this may be due to myocarditis, which in the acute setting was estimated to be present in 60% of patients with COVID-19, and more chronically is associated with more severe symptoms (4, 5). Other mechanisms, such as myocardial ischaemia, right ventricular injury (6) and postural orthostatic tachycardiac syndrome (7), may also account for chronic symptoms, whilst psychological factors are also thought to influence patient recovery following COVID-19 (3, 8).

With respect to cardiac disease, there is currently a mixed picture as to the long-term sequela following COVID-19 infection. In patients who have previously been admitted due to COVID-19, global longitudinal strain measured by echocardiography, that expresses left ventricular longitudinal shortening in percentage, and is used to assess left ventricular systolic function, was reduced as compared to controls 6 months following discharge (9), whilst left ventricular ejection fraction (LVEF) was preserved, suggesting subtle left ventricular systolic dysfunction. This is reflected by cardiopulmonary exercise testing, as a recent study by Vonbank et al. showed that patients having recovered from both mild-moderate and severe COVID-19 had cardiopulmonary exercise impairment 6 months following COVID-19, as compared to healthy controls (10).

However, at present there is limited data on cardiac sequela in patients 1 year following COVID-19 infection. A recently published meta-analysis (l2), summarizing the current literature regarding cardiac sequela in recovered COVID-19 patients, showed that there is a paucity of data from more than 6 months following acute infection with COVID-19, so the true long-term consequences are currently unknown. Equally, there is limited data on the relationship between cardiovascular symptoms following COVID-19 infection and the possible COVID-19 related cardiac involvement that may account for these symptoms. Another important limitation in our current knowledge regarding possible long-term COVID-19 related cardiac involvement is the fact that in observational studies, there are no baseline cardiovascular imaging tests available. In a recent study from the UK Biobank, where a baseline cardiovascular magnetic resonance (CMR) was available, it was shown that pre-existing adverse cardiac phenotypes were associated with greater risk of incident COVID-19 (11). Thus, the authors concluded that observational reports on cardiovascular involvement after COVID-19 may, at least partly, reflect pre-existing cardiac status rather than COVID-19 induced cardiac disease.

Given the apparent relationship between abnormal cardiac investigation results and clinical symptoms, in this study we sought to investigate the cardiac and physical performance by advanced echocardiography, CMR, and cardio-pulmonary exercise testing in patients with persistent cardiac symptoms one year following acute infection with COVID-19. We hypothesized that long-term cardiac symptoms 1 year following COVID-19 are associated with new, potentially subtle, cardiac dysfunction.

## Materials and Methods

Patients were recruited following referral to the Department of Infectious Disease at Aarhus University Hospital, Denmark. Patients were initially reviewed by the Department of Infectious Disease between August 2020 and May 2021. Patients were investigated by the Department of Cardiology between March and September 2021. All study participants were referred with long-term symptoms following a confirmed diagnosis of COVID-19, either by a SARS-CoV-2 positive PCR test during their acute illness, or an antibody test to demonstrate previous infection. Where a patient did not have a positive PCR test, the date of infection was approximated based on when a patient first displayed symptoms of SARS-CoV-2 infection. Standard investigation for dyspnoea, chest pain and palpitations were performed by the Department of Infectious Disease including blood–work, electrocardiogram (ECG), x-ray or CT thorax in case of dyspnoea, spirometry, and–in case of suspicion of pulmonary cause of dyspnea–investigation by a pulmonary physician, in order to exclude pulmonary causes of symptoms prior to referral to cardiology. In addition, anxiety and depression screening was performed, and patients were not referred for cardiology investigation if severe mental illness was identified.

Patients were offered participation in the study in case of on-going dyspnoea, chest pain and/or palpitations at the time of attendance at the long-COVID clinic and if the respiratory investigation using spirometry, electrocardiography, chest X-ray and CT thorax showed no significant pathology. To reduce the risk of including patients with pre-existing cardiac disease and thereby falsely misinterpreting any abnormal findings as COVID-19 induced cardiovascular disease, we only included a carefully selected cohort of patients without prior significant cardiovascular disease or any significant comorbidity. Additionally, patients were not referred if they had healthcare issues that would preclude full participation in the exercise investigations. Patients who had undergone echocardiography or cardio-pulmonary exercise testing prior to referral to the long-COVID clinic were also excluded to avoid unnecessary re-investigation. Assessment of cardiac function and physical performance was performed using echocardiography, cardiovascular magnetic resonance (CMR), cardio-pulmonary exercise testing, spirometry, 6-min walking test, electrocardiography and analysis of cardiac biomarkers.

### Acute Disease Symptoms

Patients were asked to report on the symptoms they experienced at the time of their acute illness. They were additionally asked if they required attendance at hospital, admission, supplementary oxygen treatment, intensive care admission or mechanical ventilation. These were corroborated using the patients' electronic patient record where possible.

### Symptoms at Study Inclusion

Patients were asked about their current symptoms at the time of their attendance at outpatient clinics at the Department of Cardiology.

### Echocardiography

Echocardiography (GE Vivid E95, Horten, Norway) was performed using a standard 2D and 3D probe. A standard clinical assessment including speckle tracking was performed. Image interpretation was based on right and left cardiac chambers structure, size and function, and evaluated in accordance with current European Association of Cardiovascular Imaging and American Society of Echocardiography guidelines (12). These results were compared to a control group. The control group consisting of 22 healthy age and sex matched individuals was obtained, with permission, from healthy participants in a previous study from Aarhus University Hospital (13).

### Cardiovascular Magnetic Resonance

CMR scanning was performed using a Philips Achieva dStream 1.5 T whole body MR scanner (Philips Medical Systems, Best, Netherlands). A standard clinical CMR protocol was performed according to consensus guidelines (14) to assess aortic flow and ventricular volumes and mass during end expiratory breath hold. T1 pre and post contrast and T2 mapping was done to visualize myocardial fibrosis and oedema, respectively. Late Gadolinium Enhancement (LGE) imaging was started 10–15 min after intravenous administration of 0.1 mmol/kg gadobutrol (Gadovist, Bayer HealthCare, Berlin, Germany). A 3D phase-sensitive inversion recovery sequence with navigator-based respiratory gating and ECG-triggering was used for LGE imaging. Subsequently, patients performed exercise testing using a CMR-compatible cycle ergometer (MRI cardiac ergometer, Lode BV, Groningen, The Netherlands).

Patients were commenced at 10 watts, progressively increasing by 10 watts/min to increase their pulse to approximately 85% of their predicted maximum pulse. A real-time cine CMR sequence with a temporal resolution of approximately 60 ms was subsequently preformed using a free breathing protocol to obtain post-exercise LV short-axis images for volumetric measurements. Post-exercise flow measurements in the aorta were then measured using breath-holding protocol to measure stroke volume. Blood pressure and pulse measurements were taken at baseline and at the end of the exercise period, prior to the CMR images being taken.

Cardiac mass and volumes were analyzed using Medviso Segment v3.2 (15). Left ventricular measurements were calculated using the fully automated 3D segmentation tool, with subsequent minimal manual adjustment. Right ventricular measurements were calculated using the semi-automatic segmentation tool, with subsequent manual adjustment when needed. Cardiac output was calculated by measurement of flow in the sinu-tubular junction times the heart rate. Circumferential, radial and longitudinal strain measurements were obtained using feature tracking CMR (FT-CMR) (16). T1 and T2 maps were assessed using Phillips Intellispace. The average T1 values and extracellular volume fractions were calculated as the mean of the 2 most basal measurements. Analysis was performed by a researcher (GW) experienced in CMR analysis. Normal values were obtained from Kawel–Boehm et al. (17).

### Cardio-Pulmonary Exercise Testing

Cardio-pulmonary exercise testing was performed using a cycle ergometer (Corival CPET, Lode BV, Groningen, The Netherlands) and processed using SentrySuite v3.10 (CareFusion, Höchberg, Germany). Patients cycled against progressively increasing levels of resistance until reaching physical exhaustion. After an initial warm-up period, load was increased by 50W every 3 min. Continuous ECG, pulse oximetry, oxygen uptake and carbon dioxide output were recorded. Blood pressure was recorded at 3-min intervals. Following completion of exercise, these measurements were recorded for a further 1 min to measure recovery. Peak oxygen uptake and peak carbon dioxide output were calculated following completion of the test. Ventilatory threshold 1 (Vt1) was calculated as the point at which CO_2_ output first increased relative to O_2_ uptake, whilst ventilatory threshold 2 (Vt2) was calculated as the point at which CO_2_ output exceeded O_2_ uptake (18). Normal values were obtained from Mezzani et al. (19) and the European Respiratory Society Guidelines (20).

### 6-Minute Walking Test

A 6-min walking test was performed to assess the number of laps a patient could complete between 2 markers placed 30 m apart. Prior to the test patients sat and relaxed for 5 min. Baseline blood pressure, pulse and oxygen saturation measurements were then taken. Subsequently, patients walked continuously for 6 min, and the distance walked was recorded. Following completion of the test further blood pressure, pulse and oxygen saturation measurements was taken. Post exercise pulse/Max Pulse was also calculated, with Max pulse calculated as 220–age. Patients also self-reported their degree of breathlessness via a modified BORG dyspnea scale from 0–10. Normal values of healthy individuals, male and female, between the ages of 40 and 80 were obtained from Casanova et al. (21).

### Serum Laboratory Sampling

Serum pro-BNP, troponin T, CRP, B-leucocytes, B-thrombocytes and INR were additionally taken for assessment of biochemical changes. Normal values shown are those provided by the biochemistry laboratory at Aarhus University Hospital.

### Statistics

Statistical analysis was performed using Graphpad Prism v. 9.1.2. Gaussian distribution of variables was calculated using the D'Agostino-Pearson normality test. An echocardiography control group consisting of 22 individuals age and gender matched to the patient group was obtained from a previous study investigating healthy individuals (13) and compared using either an unpaired *t*-test or a Mann-Whitney U test as appropriate. The CMR aortic flow measurements at baseline and during exercise were compared using a paired *t*-test. Values are expressed as mean ± standard deviation or median and interquartile range (IQR) as appropriate. A *p*-value of <0.05 was considered significant.

## Results

### Demographics and Background Information

22 participants were included in the study. 19 were female. 2 additional patients (1 male and 1 female) were originally referred for investigation, however they were unable to attend and were lost to follow-up. The median age at the time of acute illness was 47 years (IQR: 41, 53). 13 participants were healthcare professionals. Patients were reviewed in the infectious disease clinic a median of 200 days (IQR: 189, 216) following their acute illness. Patients were reviewed in the cardiology clinic a median of 401 days (IQR: 368, 442) after their acute illness. 8 had a past medical history of note, of which asthma was the most common condition. No patients were current smokers, however 3 had previously smoked. Paroxysmal atrial fibrillation had occurred in 2 patients prior to developing COVID-19. Full demographic information is outlined in Table 1. All patients had been infected with SARS-CoV-2 in prior to vaccination.

**Table 1:** Table showing the background information of the patient group. “Duration of Acute Illness” was self-reported by patients at the time of their initial referral to the Department of Infectious Disease.

	**N**
Total participants	22
Female	19
Age at time of SARS-CoV-2 infection (years)	47 (IQR 41, 53)
BMI	24.95 ± 3.03
Non-danish ethnic background	2
Duration of acute illness (Days)	14 (IQR 10, 24)
Time between acute disease and long-COVID clinic (Days)	200 (189, 216)
Time between acute disease and cardiology clinic (Days)	401 (IQR 368, 442)
**Reason for cardiology referral**	
Chest pain only	8
Dypnoea only	5
Chest pain and dyspnoea	8
Palpitations only	1

### Acute Disease Severity

Eleven patients required hospital attendance. 4 were admitted for at least 24 h. Two patients required supplementary oxygen treatment. 1 was admitted to the intensive care department, although mechanical ventilation was not required. The average duration of acute illness was 14 days (IQR 10, 24).

### Symptoms

**Figure 1:**
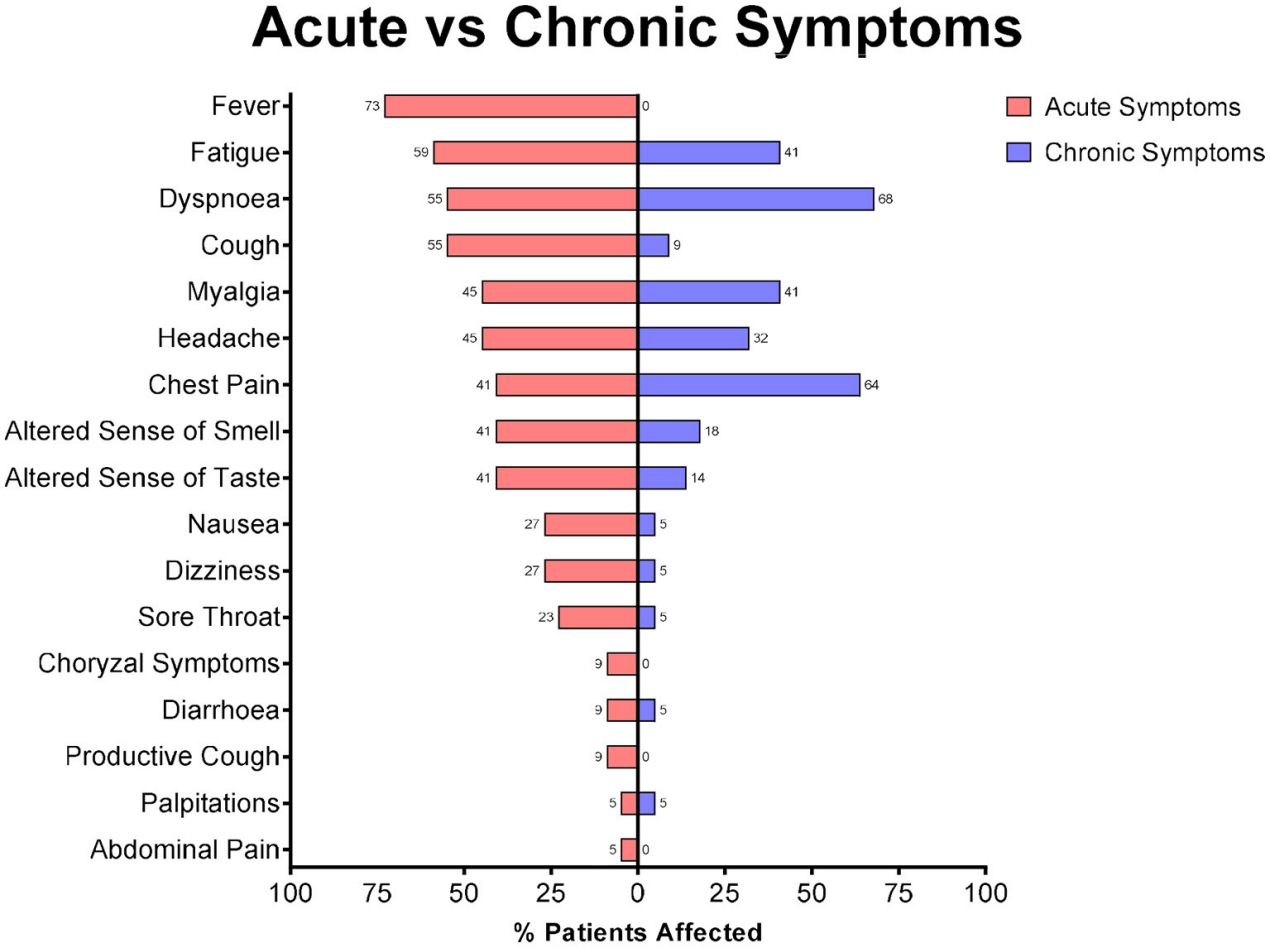
Reported symptoms of patients during the acute illness and on review in the cardiology clinic.

At the time of acute infection, the most common symptoms were fever, fatigue, cough and dyspnoea. Nine experienced chest pain and 1 complained of palpitations. At the time of review in the cardiology clinic 15 reported of dyspnoea, 14 complained of chest pain and 1 was experiencing palpitations. Of those complaining of dyspnoea, 14 were classified as NYHA class 2 and 1 was classified as NYHA class 3. Seven patients who had reported dyspnoea at the time of their review by the Department of Infectious Disease no longer experienced dyspnoea (NYHA class 1), but still reported other symptoms of cardiac disease. Of those who reported chest pain, all reported chest pain atypical for cardiac disease. A summary of the symptoms experienced by patients is shown in Figure 1.

### Acute Illness Treatment

Three patients were recorded as receiving medical treatment. All 3 received IV fluid therapy. One patient received systemic antibiotic and antiviral treatment, whilst one other was treated with systemic anti-fungal therapy. One patient required oxygen treatment, lasting 2 days.

### Blood Biochemistry

At the time of acute illness 10 patients had blood tests taken. Of those blood tests recorded, CRP on average exceeded the normal range (16.71 ± 21.14), however otherwise there were no abnormalities of note. At the time of the cardiology investigations there were no abnormalities of note. These results are shown in Tables 2, 3.

**Table 2:** Table showing the results for blood serology at the time of acute illness. Brackets contain normal values.

	**N**	
Troponin I (3-47 ng/l)	9	4.67 ± 2.45
Pro-B-Natriuretic Protein (50-300 ng/l)	5	60.20 ± 20.13
C Reactive Protein (4-8 mg/l)	10	16.71 ± 21.14
B-leucocytes (3.50-10.0 x 10^9^/l)	10	6.00 ± 2.38
B-thrombocytes (165-400 x 10^9^/l)	10	202.00 ± 49.51
INR (1-1.2)	8	1.06 ± 0.05

**Table 3:** Table showing the results for blood serology at the time of investigation for long-term cardio-pulmonary symptoms. Brackets contain normal values.

	**N**	
Troponin I (3–47 ng/l)	22	5.59 ± 6.59
Pro-B-Natriuretic Protein (50–300 ng/l)	22	88.18 ± 54.27
C Reactive Protein (4–8 mg/l)	22	5.25 ± 3.50
B-leucocytes (3.50–10.0 x 10^9^/l)	22	7.27 ± 2.55
B-thrombocytes (165–400 x 10^9^/l)	22	266.70 ± 64.15
INR (1–1.2)	22	1.05 ± 0.06

### Electrocardiography

21 of the 22 ECG recordings were assessed as normal. One patient with known untreated hypertension had ECG signs of left ventricular hypertrophy. As such hypertension was diagnosed as the cause. There was no evidence of myocardial ischaemia or pericarditis on any ECG.

### Echocardiography

LVEF was normal (61.45 ± 6.59%). Mean Global Longitudinal Strain (GLS)% was 19.80 ± 3.12%, however 7 were abnormal (i.e., <18%). Tricuspid annular plane systolic excursion (TAPSE) was 24.95 ± 5.55 mm. Three patients had a Mitral Valve Insufficiency Grade (MI grade) 1, 1 had an Aortic Valve Insufficiency Grade (AI grade) 1 and 6 had a Tricuspid Valve Grade (TR grade) 1. No patients had evidence of pericardial effusion. All other results were within the normal range (12).

As compared to the healthy control group, E/A ratio (1.67 ± 0.33 vs. 1.32 ± 0.44, *p* = 0.006), E-DT (204.07 ± 44.18 vs. 174.77 ± 32.79 ms, *p* = 0.016) and E/é (8.32 ± 2.44 vs. 5.19 ± 1.47, *p* = <0.001) were significantly smaller than the control group. There were no differences in LVEF (63.03 ± 4.05 vs. 61.45 ± 6.59, *p* = 0.344) or GLS (20.70 ± 1.84 vs. 19.80 ± 3.12, *p* = 0.253). Full echocardiography results are shown in Supplementary Table 1.

### Baseline Cardiac Magnetic Resonance

All measurements of cardiac mass and volume at baseline were within the normal range. LVEF was measured at 62.36 ± 4.01 %, whilst right ventricular ejection fraction (RVEF) was 52.59 ± 8.98%. All volumetric measurements were also normal once corrected for the patients' body surface area. Full results are shown in Supplementary Table 2.

### Exercise Cardiovascular Magnetic Resonance

There was a statistically significant increase in both heart rate (70.18 ± 10.64 vs. 105.30 ± 15.10 bpm, *p* = <0. 001) and cardiac output (5.19 ± 1.04 vs. 7.32 ± 1.93 l/min, *p* = <0. 001) with exercise compared to baseline. Stroke volume did not change (81.78 ± 14.45 vs. 82.90 ± 18.25 ml, *p* = 0.763). LV end-diastolic volume significantly decreased (138.00 ± 20.61 v.s 121.30 ± 23.76 ml, *p* = <0.001). There was no change in LVEF (62.36 ± 4.01 vs. 64.65 ± 5.96 %, p = 0.126). All LV BSA adjusted and non-adjusted values lay within the normal range (17).

Right ventricular end diastolic volume (156.00 ± 29.76 vs. 124.90 ± 27.87 ml, *p* = <0.001) and end systolic volume (74.84 ± 22.07 vs. 52.89 ± 16.53 ml, *p* = <0.001) were significantly reduced, whilst there was a small reduction in stroke volume (81.19 ± 15.81 vs. 71.98 ± 18.31 ml, *p* = 0.034). RVEF increased with exercise (52.29 ± 8.98 vs. 57.64 ± 9.45 %, *p* = 0.033). All other values lay within the normal range. Full results are shown in Supplementary Table 2.

### Cardiac Strain Measurements

At baseline, LV peak mean circumferential strain (20. 65 ± 3.59), peak mean radial strain (48.80 ± 11.32) and peak mean longitudinal strain (17.19 ± 3.48) were within the normal range. RV peak mean circumferential strain was also normal (10.33 ± 4.41). Full results are shown in Supplementary Table 3.

### T1 Maps/Late Gadolinium Enhancement (LGE)

There was evidence of late gadolinium enhancement in the lateral wall of the left ventricle in 1 patient from the cohort. There was otherwise no visual evidence of contrast enhancement on the LGE images. The native T1 values were normal (1043.43 ± 38.77 msec) and ECV was also normal, with a value of 28.60 ± 2.25, suggesting the absence of diffuse myocardial fibrosis. T2 was normal (51.96 ± 2.14), indicating there was no oedema within the myocardium. Normal values were obtained from Kawel–Boehm et al. (17). Full results are shown in Supplementary Table 4.

### 6-Minute Walking Test

The mean distance walked was 608.9 ± 54.51 m, exceeding the average of 571 ± 90m reported by Casanova et al. (21). There was a small but significant increase in MAP post-exercise (95.98 ± 11.77 mmHg vs. 100.20 ± 12.41 mmHg, *p* = 0.002), and a more substantial increase in pulse (73.05 ± 13.02 bpm vs. 91.95 ± 16.71 bpm, *p* = <0.001). There was no significant change in O2 saturations pre-and post-exercise (98.55 ± 1.18 % vs. 98.27 ± 1.08 %, *p* = 0.110). Post-exercise pulse/Max Pulse was 52.98 ± 10.05 %, less than the normal value of 73 ± 13%. Median post-exercise BORG scale measurement was 2.50 (IQR: 2.00, 3.00), equating to slight-moderate breathlessness. This was substantially greater than the median value of 0.5 (IQR: 0, 3) reported by Casanova et al. (21). Full results are available in Supplementary Table 5.

### Cardio-Pulmonary Exercise Test

**Figure 2:**
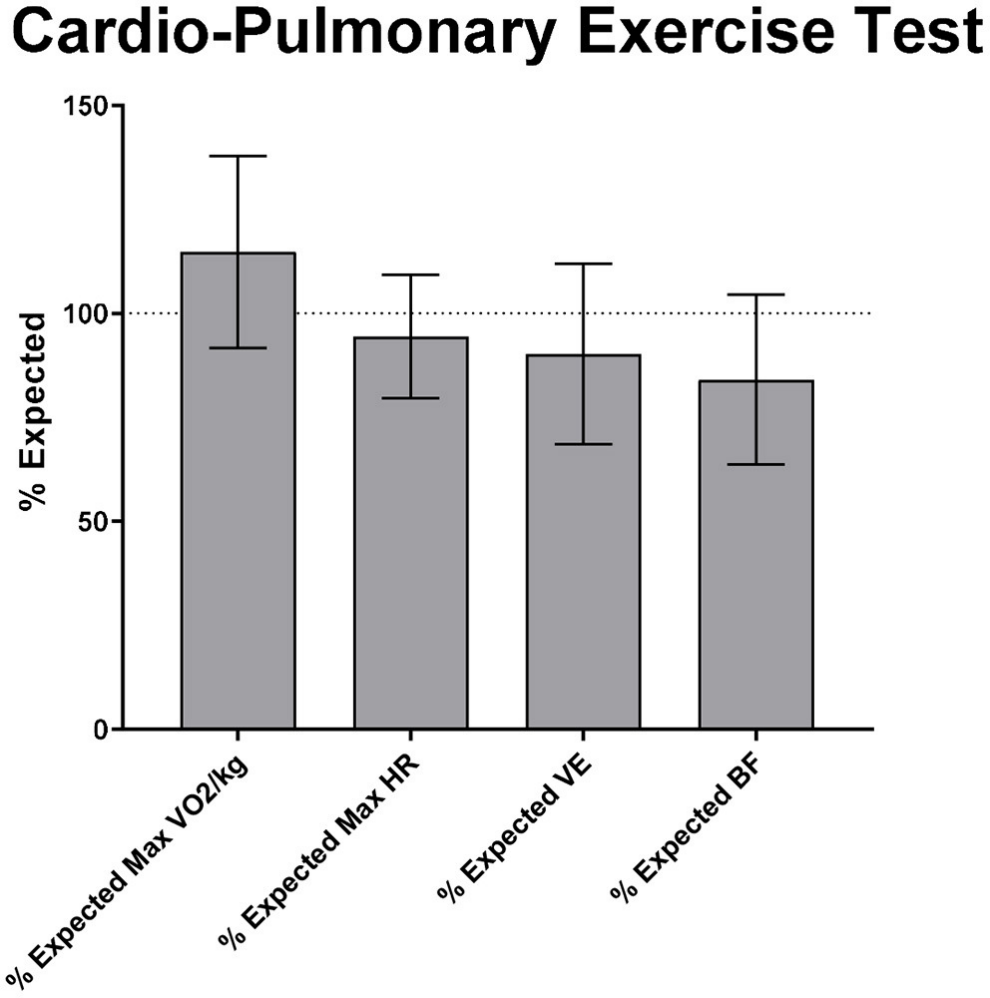
Cardio-pulmonary exercise test results as % expected for the patients' age, sex and ethnicity. HR, heart rate; VE, minute ventilation; BF, breathing frequency.

VO2 max was 2045 ± 658.40 ml/min. Respiratory Exchange Ratio (RER) was 1.14 ± 0.11, exceeding the 1.10 threshold for normal RER. Metabolic Equivalents (METS) was 8.26 ± 2.12. Patients on average exceeded their % expected resistance (132.8 ± 34.41%), % expected max VO2 (116.2 ± 24.11%) and % expected VO2/kg (116.2 ± 24.11%). Average % expected ventilation (VE) (91.72 ± 22.95%) and % expected breathing frequency (BF) (84.33 ± 21.12%) were less than expected for the patients' age and weight. Vt1 was normal as a proportion of VO2 max (40.24 ± 8.61 %), whilst Vt2 was >50% (68.06 ± 12.48 %), indicating a normal anaerobic threshold (20). A summary of the % expected values is shown in Figure 2. Full results are available in Supplementary Table 6.

## Discussion

To our knowledge, this is the first study to have investigated patients with cardiac symptoms 1 year following infection with SARS-CoV-2. Our results show that in a cohort of patients reporting long-term cardiac symptoms following COVID-19, there was no impairment in their exercise capacity as compared to that which would be expected for their age, gender and ethnicity, as demonstrated by a normal VO2 max and 6-min walking test. LVEF and GLS as measured by both echocardiography and CMR were in the normal range in this cohort as a whole.

Cardio-pulmonary exercise testing showed that this cohort on average exceeded the exercise capacity that would be expected for their age, gender and ethnicity. This is demonstrated by the percent predicted peak VO2 being 114.8% on average, indicating that the cardiac output is likely to be normal. The percent predicted O2 pulse, calculated as VO2/heart rate ratio and reflecting the quantity of oxygen consumed per heartbeat (22), was on average 123%. This indicates that the heart was consuming oxygen efficiently. This is further supported by a mean METS value of 8.26, which demonstrates that the patients were able to undergo strenuous exercise (22). Additionally, the maintenance of ejection fraction whilst LV and RV EDV and ESV values were reduced following exercise, mirrors the findings of Alegret et al. (23), who found the same response in healthy individuals using a static exercise CMR protocol.

Furthermore, there was no statistically significant desaturation during the 6-min walking test, indicating that patients were able to complete normal activities without a measurable decline in saturation. Previous research has found a reduced VO2 peak as a consequence of COVID-19 infection (24, 25), however these studies have focused on patients that have previously been admitted to hospital and reviewed subsequently regardless of their symptoms, as opposed to this study that assessed patients according to their chronic symptoms, regardless of the severity of their acute COVID-19 infection.

Global longitudinal strain as assessed by echocardiography and CMR was within the normal range. This could suggest that the reduction in GLS seen at 3 months (26, 27) and 6 months (9) in those admitted to hospital with COVID-19 may have resolved 1 year following their acute disease. This could mean that the abnormal LV parameters recover at a slower rate, as compared to RV function (26). This supports the findings of Gorecka et al., who equally found no statistically significant differences in cardiac structure, function, or tissue characterization, compared to a healthy control group (28).

With respect to the one individual that had signs of late gadolinium enhancement in the lateral wall of the LV, the patient reported that she was a regular runner, who previously had run ultra-marathons. Intensive exercise has previously been described as a cause of this pattern of late gadolinium enhancement (29). Given that her exercise testing was otherwise normal, it is more likely that the late gadolinium enhancement was due to rigorous exercise.

The lack of any increase in Nt-proBNP or troponins supports the normal findings of an overall normal systolic function assessed by both echocardiography and CMR. These findings are in accordance with Kim et al., who in a recent meta-analysis reported that normal cardiac enzymes were predictive of a lower prevalence of abnormal CMR findings in recovered COVID-19 patients (30).

The patients' cardiac symptoms were all self-reported symptoms. We did not find any evidence of cardiac dysfunction nor did we find any major reductions in cardiopulmonary exercise capacity that could substantiate or explain these symptoms. However, the post-exercise BORG scale measurement after the 6-min walking test did reveal that the patients felt slight-moderate breathlessness, which is not expected in healthy subjects. Thus, in this patient cohort, there seems to be a discrepancy between the patients' subjective feeling of breathlessness and the actual measured cardiovascular fitness. The reason for this is unclear, One suggestion for this may be that the sensation of exhaustion does not occur due to issues with cardio-respiratory function. A recent study from our research group showed that in a group of 20 patients with persistent neuromuscular symptoms, including fatigue, 11 had myopathic changes, as assessed using quantitative electromyography (31).

Another reason for this discrepancy may be, in part, because of psychological stress following COVID-19. Data from Huang et al. (3, 8) showed that 23% and 26% of patients experienced anxiety of depression at 6 and 12 months following COVID-19 respectively, which only slightly altered with increasing acute disease severity. Furthermore, women were significantly more likely to report symptoms of anxiety or depression, which, given the larger number of women in our cohort, may account for the discrepancy in reported symptoms vs. measured cardiovascular fitness in our study. In our cohort, individuals with obvious signs of anxiety or depression were not referred to the cardiology department, however we did not perform a systematic psychiatric evaluation in our study, so further investigation is required to determine any relationship between psychological factors and reported symptoms.

One limitation of this study is the relative homogeneity of the study group, which is predominantly female and predominantly of western Caucasian ethnic background. Furthermore, this cohort contained a disproportionate amount of healthcare workers, however this may reflect the increased risk that healthcare workers experienced of becoming infected with SARS-CoV-2 during the initial phase of the pandemic in Denmark. Another limitation is that patients who had previously been referred for echocardiography or CPET were excluded from participation in this study. This was to avoid duplicate investigation. However, there is a possibility that some individuals with cardiac dysfunction could have been refused inclusion in this study. A further limitation is the absence of a more extensive psychological assessment of patients included in this study, which may have assisted in drawing more certain conclusions regarding the patients' subjective experience of symptoms. As stated previously, future investigations may benefit from considering these factors as a part of their protocol. Finally, this study had a small cohort of 22 individuals. However, these individuals have been investigated very extensively, using multiple modalities. Furthermore, the careful selection of the patient cohort was intended to include individuals without significant comorbidity including prior known cardiovascular disease, since we sought to determine whether any specific presumed COVID-19 related cardiac pathology could be identified without the risk of falsely including pre-existing cardiac abnormalities.

In conclusion, in a cohort of 22 patients without significant comorbidity experiencing symptoms suggestive of cardiac disease for 1 year following COVID-19 infection, there was no evidence of significant cardiac abnormalities from echocardiography or CMR. Furthermore, cardiovascular fitness evaluated by cardio-pulmonary exercise testing was normal. Our hypothesis, that cardiac dysfunction accounts for symptoms of cardiac disease in patients 1 year following COVID-19, is not supported by the findings of our study. This suggests that among otherwise healthy subjects without prior significant cardiac disease, long-term dyspnoea, chest pain or palpitation following COVID-19 may not be attributed to underlying COVID-19 related cardiac disease.

## Data Availability Statement

The original contributions presented in the study are included in the article/Supplementary Material, further inquiries can be directed to the corresponding author.

## Ethics Statement

The studies involving human participants were reviewed and approved by The Central Denmark Region Regional Ethics Committee. The patients/participants provided their written informed consent to participate in this study.

## Author Contributions

GW, JA, SL, EH, CL, HJ, LØ, HB, SP, and WK contributed to the conception of the study. GW, WK, SL, and SP designed the study protocol. GW, TK, JA, AL, and SP performed data acquisition. GW, TK, and WK performed data analysis. GW, WK, SL, and JA drafted the article. All authors have reviewed and approved the manuscript.

## Funding

This study was founded by a grant from the Novo Nordisk Foundation (NNF21OC0066984). WK was funded by the Health Research Foundation of Central Denmark Region (A2573).

## Conflict of Interest

The authors declare that the research was conducted in the absence of any commercial or financial relationships that could be construed as a potential conflict of interest.

## Publisher's Note

All claims expressed in this article are solely those of the authors and do not necessarily represent those of their affiliated organizations, or those of the publisher, the editors and the reviewers. Any product that may be evaluated in this article, or claim that may be made by its manufacturer, is not guaranteed or endorsed by the publisher.
