# Supplementary material for: Cardiac Performance and Cardiopulmonary Fitness After Infection With SARS-CoV-2
Source: Front Cardiovasc Med. 2022 May 13;9:871603. doi: 10.3389/fcvm.2022.871603 (PMC9136046; doi:10.3389/fcvm.2022.871603)
Supplement: Supplementary file 1 [file Data_Sheet_1.pdf]

Supplementary Files

Supplementary Table 1

Table showing the results for echocardiography, alongside with a healthy control group, where those values were available. Brackets contain normal values. LAVI values were not available for 2 controls, as well as to 2 patients. 1 individual had breast implants which resulted in poor visualisation of the heart. IVS = Inter-Ventricular Septal Thickness, PW = Posterior Wall Thickness, LVEDD = Left Ventricular End Diastolic Diameter, LVESD = Left Ventricular End Systolic Diameter, LVEDV = Left Ventricular End Diastolic Volume, LVESV = Left Ventricular End Systolic Volume, LVEF = Left Ventricular Ejection Fraction, LVOT-VTI = Left Ventricular Outflow Tract Velocity Time Integral, GLS = Global Longitudinal Strain, LA = Left Atrium, LAVI = LA Volume Index, LAEF = LA Ejection Fraction, TAPSE = Tricuspid Annular Plane Systolic Excursion, TR = Tricuspid Valve Regurgitation, VCI = Inferior Vena Cava, MI = Mitral Valve Insufficiency, AI = Aortic Valve Insufficiency. Normal values are obtained from Lang et al. (12) and are shown in brackets.

	**N**	**Patient**	**Control**	**P Value**
IVS (6 – 10 mm)	21	7.48 ± 1.54	7.85 ± 1.23	0.393
PW (6 – 10 mm)	21	6.91 ± 1.30	6.61 ± 2.95	0.672
LVEDD (38 – 58 mm)	21	47.43 ± 4.51	47.22 ± 6.32	0.902
LVESD (22 – 40 mm)	21	30.19 ± 4.78	29.82 ± 5.14	0.810
LVEDV (46 – 150 ml)	22	90.41 ± 27.99	104.18 ± 17.93	0.059
LVESV (14 – 61 ml)	22	37.91 ± 16.10	38.45 ± 7.93	0.452
LVEF (52 – 74 %)	22	61.45 ± 6.59	63.03 ± 4.05	0.344
LVOT-VTI (cm/s)	21	22.93 ± 4.55	24.22 ± 3.45	0.306
GLS (> 18%)	22	19.80 ± 3.12	20.70 ± 1.84	0.253
LA volume (ml)	20	48.15 ± 10.04	55.20 ± 15.11	0.090
LAVI (16 – 34 ml/m2)	18	26.86 ± 5.28	31.20 ± 9.16	0.351
LAEF (%)	16	47.56 ± 11.25	-	-
E/A ratio (0.8 – 2.0)	22	1.32 ± 0.44	1.67 ± 0.33	***0.006***
E-DT (119 – 242 ms)	22	174.77 ± 32.79	204.07 ± 44.18	***0.016***
E/é (<6)	22	5.16 ± 1.47	8.32 ± 2.44	***<0.001***
TAPSE (>17 mm)	22	24.95 ± 5.55	25.90 ± 6.88	0.617
S' cm/s	22	14.32 ± 1.94	-	-
TR gradient mmHg	12	16.53 ± 7.62	-	-
VCI max (<21 mm)	21	15.71 ± 3.52	-	-
VCI min (mm)	21	5.05 ± 4.47	-	-
MI grade 1	22	3	-	-
AI grade 1	22	1	-	-
TR grade 1	22	9	-	-

Supplementary Table 2

Table showing the results for CMR volume measurements at baseline compared with post exercise measurements. RV = Right Ventricle. Normal values at baseline were obtained from Kawel - Boehm et al. (17) and are shown in brackets.

	N	Baseline	Exercise	P Value
Heart Rate (60 – 100 bpm)	22	70.18 ± 10.64	105.30 ± 15.10	***<0.001***
Left Ventricular Mass (41 – 176 g)	22	83.85 ± 20.68	84.92 ± 18.47	0.552
Left Ventricular Mass Index (30 – 85 g/m2)	22	46.19 ± 8.09	46.83 ± 6.94	0.521
End Diastolic Volume (78 – 215 ml)	22	138.00 ± 20.61	121.30 ± 23.76	***<0.001***
End Diastolic Volume Index (50 – 108 ml/m2)	22	76.91 ± 10.66	67.41 ± 11.98	***<0.001***
End Systolic Volume (21 – 85 ml)	22	52.24 ± 11.31	43.25 ± 13.06	***0.002***
End Systolic Volume Index (10 – 47 ml/m2)	22	29.04 ± 5.61	23.91 ± 6.33	***<0.001***
Stroke Volume (52 – 145 ml)	22	85.77 ± 11.71	78.03 ± 14.93	***0.020***
Stroke Volume Index (33 – 72 ml/m2)	22	47.87 ± 6.68	43.50 ± 8.61	***0.021***
Ejection Fraction (44 – 79 %)	22	62.36 ± 4.01	64.65 ± 5.96	0.126
Cardiac Output (2.7 – 7.8 l/min)	22	6.01 ± 1.16	8.12 ± 1.51	***<0.001***
Cardiac Index (1.8 – 4.2 l/min/m^2)	22	3.35 ± 0.65	4.53 ± 0.85	***<0.001***
Peak Filling Rate (185 – 807 ml/s)	22	582.00 ± 159.90	446.20 ± 122.30	***0.001***
Peak Ejection Rate (ml/s)	22	654.10 ± 138.50	554.20 ± 147.10	***0.022***
RV End Diastolic Volume (68 – 244 ml)	22	156.00 ± 29.76	124.90 ± 27.87	***<0.001***
RV End Diastolic Volume Index (48 – 123ml/m2)	22	86.81 ± 14.84	69.42 ± 14.64	***<0.001***
RV End Systolic Volume (20 – 117 ml)	22	74.84 ± 22.07	52.89 ± 16.53	***<0.001***
RV End Systolic Volume Index (13 – 59 ml/m2)	22	41.40 ± 11.16	29.32 ± 8.93	***<0.001***
RV Stroke Volume (39 – 146 ml)	22	81.19 ± 15.81	71.98 ± 18.31	***0.034***
RV Stroke Volume Index (28 – 75 ml/m2)	22	45.41 ± 9.10	40.11 ± 10.12	***0.027***
RV Ejection Fraction (42 – 74 %)	22	52.59 ± 8.98	57.64 ± 9.45	***0.033***
RV Peak Filling Rate (ml/s)	22	725.60 ± 265.50	405.20 ± 143.80	***<0.001***
RV Peak Ejection Rate (ml/s)	22	735.80 ± 192.90	525.10 ± 158.30	***<0.001***

Supplementary Table 3

Table showing the results for CMR flow and strain measurements. These two sub-groups were compared to assess for any significant differences. LV = Left Ventricle, RV = Right Ventricle. Normal values are obtained from Kawel- Boehm et al. (17) and are shown in brackets. Neither LV Mean Radial Strain nor RV Mean Circumferential Strain normal values were not available.

	N	Baseline	Exercise	P Value
Total Flow Volume (52 – 145 ml)	22	81.78 ± 14.45	82.90 ± 18.25	0.763
Total Flow (2700 – 7800 ml/min)	22	5190 ± 1042	7319 ± 1927	***<0.001***
Heart Rate (60 – 100 bpm)	22	63.86 ± 9.25	89.87 ± 19.22	***<0.001***
LV Mean Circumferential Strain (14.6 – 29.2)	22	20.65 ± 3.59	-	-
- Basal	22	19.02 ± 3.94	-	-
- Mid	22	18.13 ± 3.13	-	-
- Apical	22	25.10 ± 5.41	-	-
LV Mean Radial Strain	22	48.80 ± 11.32	-	-
- Basal	22	42.37 ± 13.14	-	-
- Mid	22	54.23 ± 13.23	-	-
- Apical	22	49.84 ± 14.28	-	-
RV Mean Circumferential Strain	22	10.33 ± 4.41	-	-
LV Mean Longitudinal Strain (12.7 – 28.7)	22	17.19 ± 3.48	-	-

Supplementary Table 4

Table showing the results for T1/T2 maps and Late Gadolinium Enhancement. 3 patients did not receive a contrast agent as they had previously undergone CMR, and as such it was considered not clinically justified to repeat the investigation. ECV = Extracellular Volume. Normal ranges are obtained from Kawel-Boehm et al. (17) and are shown in brackets.

	**N**	
T1 Native (905 - 1073 ms)	20	1050.00 ± 52.20
T1 Enhanced (415 ± 113 ms)	19	472.89 ± 32.27
ECV (17 – 29 %)	19	28.60 ± 2.25
T2 Native (50 – 62 ms)	17	51.96 ± 2.14

Supplementary Table 5

Table showing the results for the 6-minute walking test. MAP = Mean Arterial Pressure, O2 Saturations. Normal values from Casanova et al. (21) and are shown in brackets.

	**N**	
Baseline MAP (mmHg)	22	95.98 ± 11.77
Baseline Pulse (76 ± 10 bpm)	22	73.05 ± 13.02
Baseline O2 Saturations (%)	22	98.55 ± 1.18
Distance Walked (571 ± 90 m)	22	608.90 ± 54.51
Post-exercise MAP (mmHg)	22	100.20 ± 12.41
Post-exercise Pulse (bpm)	22	91.95 ± 16.71
Post-exercise Pulse/Estimated Max Pulse (73 ± 10 %)	22	52.98 ± 10.05
Post-exercise O2 Saturations (%)	22	98.27 ± 1.08
Post-exercise Borg Scale (0.5, IQR: 0-3)	15	2.50 (IQR 2.00, 3.00)

Supplementary Table 6

Table showing the results for the cardio-pulmonary exercise test. Due to an error in recording, some values were not obtained. HR = Heart rate, BP = Blood pressure, O2 = Oxygen, CO2 = Carbon Dioxide, SpO2 = Oxygen Saturation, VECO2 = CO2 Minute Ventilation, VT1 = Ventilatory threshold 1, VT2 = Ventilatory threshold 2. Normal values are obtained from Mezzani (19) and the European Respiratory Society guidelines (20) and are shown in brackets. Due to the differences in patient age and gender, normal values as a percentage of predicted results rather than raw values of age and gender-dependent variables are provided.

	**N**	
Maximum Resistance (W)	22	159.10 ± 56.98
% Expected Resistance (>80%)	21	131.70 ± 32.46
Maximum Oxygen Consumption (VO2) (ml/min)	21	2045.00 ± 658.40
% Expected Max VO2 (>85%)	21	114.80 ± 23.08
Maximum Carbon Dioxide Output (VCO2) (ml/min)	21	2263.00 ± 759.50
Maximum VO2/kg/min (>20 mL/kg/min)	21	28.90 ± 7.41
% Expected VO2/kg (>85%)	21	114.80 ± 23.08
Respiratory Exchange Ratio (RER) (>1.05)	22	1.14 ± 0.11
Minute Ventilation (VE) (l/min)	21	79.62 ± 24.26
% Expected VE (>85%)	21	90.24 ± 21.74
Breathing Frequency (BF) (< 50 breaths/min)	21	37.19 ± 6.76
% Expected BF	21	84.10 ± 20.40
Metabolic Equivalents (METS)	21	8.26 ± 2.12
Maximum Heart Rate (bpm)	21	167.20 ± 24.97
% Expected Max HR (>90%)	21	94.43 ± 14.84
O2 Pulse (VO2/HR)	20	12.32 ± 3.77
% Expected O2 Pulse (>80%)	20	123.00 ± 25.88
End-exercise Systolic BP (mmHg)	21	176.90 ± 29.16
End-exercise Diastolic BP (mmHg)	21	98.48 ± 23.82
End-exercise SpO2 (>95%)	20	98.70 ± 2.00
VECO2s (<40)	21	30.03 ± 3.93
VT1 (mLO_2/Kg/min)	22	11.52 ± 1.80
VT1 as % VO_2 max (>40%)	21	40.24 ± 8.61
VT2 (mLO_2/Kg/min)	22	19.83 ± 5.51
VT2 as % VO_2 max (>50%)	21	68.06 ± 12.48
